# Concentration of Thyrotropic Hormone in Persons Occupationally Exposed to Lead, Cadmium and Arsenic

**DOI:** 10.1007/s12011-017-1096-x

**Published:** 2017-07-19

**Authors:** Marta Jurdziak, Paweł Gać, Małgorzata Poręba, Anna Szymańska-Chabowska, Grzegorz Mazur, Rafał Poręba

**Affiliations:** 1 Department of Internal Medicine, Occupational Diseases and Hypertension, Wroclaw Medical University, Borowska 213, 50-556 Wroclaw, Poland; 2 Department of Hygiene, Wroclaw Medical University, Mikulicza-Radeckiego 7, 50-368 Wroclaw, Poland; 3 Department of Pathophysiology, Wroclaw Medical University, Marcinkowskiego 1, 50-368 Wroclaw, Poland

**Keywords:** Lead, Cadmium, Arsenic, Occupational exposure, Thyroid, Thyrotropic hormone

## Abstract

Thyroid hormones are essential for body homeostasis. The scientific literature contains restricted proofs for effects of environmental chemical factors on thyroid function. The present study aimed at evaluating the relationship between toxicological parameters and concentration of thyrotropic hormone in persons occupationally exposed to lead, cadmium and arsenic. The studies were conducted on 102 consecutive workers occupationally exposed to lead, cadmium and arsenic (mean age 45.08 ± 9.87 years). The estimated parameters characterizing occupational exposure to metals included blood cadmium concentration (Cd-B), blood lead concentration (Pb-B), blood zinc protoporphyrin concentration (ZnPP) and urine arsenic concentration (As-U). Thyroid function was evaluated using the parameter employed in screening studies, the blood thyrotropic hormone concentration (TSH). No differences were disclosed in mean values of toxicological parameters between the subgroup of persons occupationally exposed to lead, cadmium and arsenic with TSH in and out of the accepted normal values. Logistic regression demonstrated that higher blood total bilirubin concentrations (ORu = 4.101; *p* = 0.025) and higher Cd-B (ORu = 1.532; *p* = 0.027) represented independent risk factors of abnormal values of TSH in this group. In conclusion, in the group of workers exposed to lead, cadmium and arsenic, higher blood cadmium concentration seems to augment the risk of abnormal hormonal thyroid function.

## Background

Thyroid hormones are essential for body homeostasis and functioning of nervous, cardiovascular and reproductive systems, and they control body growth [1–3]. Their involvement remains particularly significant in development of the central nervous system (CNS), particularly of neurons in the cerebral cortex, and in development of nervous projections, their myelinization and correct blood supply in CNS [2]. Thyroid function is controlled by the hypothalamic-pituitary- thyroid axis, with mediation of thyroliberin (TRH), thyrotropic hormone (TSH), thyroxine (T4) and triiodothyronine (T3), linked by a complex network of negative feedbacks. Abnormalities in thyroid function represent a frequent pathology. Hyperthyroidism affects around 1.6% women and 0.14% men, and it ranges from 1 to 6% individuals up to 60 years of age (fivefold more frequently in women). Normal thyroid function depends on bioavailability of several trace elements, mainly iodine, providing a substrate for production of thyroid hormones and selenium (a significant element of deiodinases) but also of iron, zinc and copper [4].

The available scientific literature contains restricted proofs for effects of environmental chemical factors on thyroid function. Respective studies were conducted on polychlorinated biphenyls (PCB), phthalates, perfluorinated compounds and metals, including arsenic, cadmium, lead, manganese and mercury [5–15]. However, the current studies on the topic brought no unequivocal results.

The present study aimed at evaluating the relationship between toxicological parameters and thyrotropic hormone concentration (the screening index of thyroid function) in persons occupationally exposed to lead, cadmium and arsenic.

## Methods

The studies were conducted during prophylactic tests in a group of 102 consecutive smelting workers who met the inclusion criteria for the project. The inclusion criteria were as follows: occupational exposure to lead, cadmium and arsenic, the total duration of employment with the exposure to metals amounting to at least 1 year and, simultaneously, the absence of the current exposure to other chemical or physical substances documented by the Safety and Hygiene section. The size and quality of the environmental exposure was similar for all of the workers included into the study. All persons inhabited the same region, at similar distances to the road traffic. The study group included only subjects who inhabited the region for a longer time (at least for 10 years). Clinical characteristics of the study group are presented in Table 1.

**Table 1:** Clinical characteristics of the study group

	Mean	Median	Standard deviation	Minimum	Maximum
Age (years)	45.08	46.00	9.87	25.00	63.00
Height (m)	1.75	1.76	0.08	1.59	1.93
Body mass (kg)	90.67	90.00	19.71	51.00	140.00
Body mass index (kg/m^2^)	29.34	29.60	5.23	18.30	45.20
Body surface area (m^2^)	2.06	2.04	0.24	1.55	2.63
Waist circumference (cm)	102.08	102.00	14.93	67.00	138.00
	*n*		%		
Gender					
Men	83		81.4		
Women	19		19.6		
Smoking	45		44.12		
Overweight/obesity	75		73.5		
Overweight	29		28.4		
Obesity	46		45.1		
Arterial hypertension	43		42.2		
Coronary artery diseases	4		3.9		
Dyslipidaemia	9		8.8		
Diabetes	11		10.8		

Basing on anamnesis and physical examination carried out in all individuals taking part in the study, personal and general information was obtained related to health condition and occupational exposure to metals.

In every participant, anthropometric measurements were conducted. Then for estimation of laboratory tests, 25 ml of venous blood and standard urine sample from each worker right after finishing work was collected. The laboratory tests included blood cell count and conventional biochemical tests detecting serum creatinine, uric acid, aminotransferases, bilirubin, glucose, cholesterols, triglycerides, potassium and sodium. Thyroid function was evaluated using the parameter employed in screening studies, the blood thyrotropic hormone concentration (TSH). The laboratory characteristics are presented in Table 2.

**Table 2:** Laboratory parameters in the study group

	Mean	Median	Standard deviation	Minimum	Maximum
White blood cells (G/l)	7.44	7.33	1.64	4.62	14.32
Red blood cells (T/l)	4.98	4.92	0.36	4.13	5.73
Haemoglobin (g/dl)	15.10	15.30	1.12	11.50	18.30
Platelet count (G/l)	235.58	236.50	60.13	56.00	431.00
Total bilirubin (mg/dl)	0.63	0.60	0.26	0.20	1.60
Alanine aminotransferase (U/l)	36.72	32.50	17.70	9.00	93.00
Aspartate aminotransferase (U/l)	31.21	28.50	15.63	13.00	141.00
Glucose (mg/dl)	102.36	91.00	36.66	63.00	299.00
Creatinine (mg/dl)	1.07	1.08	0.15	0.80	1.65
eGFR (ml/min)	76.25	74.50	11.45	45.00	105.00
Uric acid (mg/dl)	6.28	6.40	1.48	2.90	9.70
Total cholesterol (mg/dl)	232.85	230.50	56.61	87.00	539.00
High-density lipoprotein (mg/dl)	48.44	47.00	12.51	27.00	89.00
Low-density lipoprotein (mg/dl)	138.39	138.00	39.26	24.00	237.00
Triglycerides (mg/dl)	262.71	178.00	253.21	50.00	1513.00
TSH (mIU/l)	2.18	1.96	1.13	0.55	6.19
Potassium (mmol/l)	4.79	4.74	0.42	3.73	5.91
Sodium (mmol/l)	139.21	139.00	2.14	131.00	144.00

*eGFR* estimated glomerular filtration rate, *TSH* thyrotropic hormone

The examined parameters characterized occupational exposure to metals including blood cadmium concentration (Cd-B), blood lead concentration (Pb-B), blood zinc protoporphyrin concentration (ZnPP) and urine arsenic concentration (As-U). In addition, microelements were estimated, including blood copper concentrations (Cu-B) and blood zinc concentration (Zn-B). Cd-B and Pb-B were measured by graphite furnace atomic absorption spectrometry Solaar M6 of Thermo Elemental, UK. As-U was measured by hydride generation atomic absorption spectrometry (HGAAS) using the VP100 Continuous Flow Vapour System. Cu-B and Zn-B were determined by flame atomic absorption spectrometry (Thermo Elemental Solaar M6, UK). ZnPP erythrocyte concentration was measured using a rapid, fluorometric screening method by means of a ProtoFluor hematofluorometer by Helena (USA). Concentrations of metals and metalloid in samples of blood or urine in the study group are shown in Table 3.

**Table 3:** Concentrations of metals and metalloid in samples of blood or urine in the study group

	Mean	Median	Standard deviation	Minimum	Maximum
Duration of work with exposure (years)	16.34	14.00	11.53	1.00	43.00
Cu-B (mg/dl)	111.50	110.50	18.27	84.00	139.00
Zn-B (mg/dl)	84.42	81.60	9.70	71.90	99.90
Cd-B (μg/l)	2.34	0.71	3.16	0.34	8.23
As-U (μg/g crea)	14.31	10.90	12.51	2.43	89.50
Pb-B (μg/l)	222.09	222.00	140.98	16.60	638.00
ZnPP (μg/dl)	42.67	34.00	28.94	17.00	203.00

*As-U* concentration of arsenic in urine, *Cd-B* concentration of cadmium in blood, *Cu-B* concentration of copper in blood, *Pb-B* concentration of lead in blood, *Zn-B* concentration of zinc in blood, *ZnPP* concentration of zinc protoporphyrin in blood

In subsequent stages of the study, using as cutoff levels normative values of TSH (reference range of 0.4–4.0 mIU/l), in persons occupationally exposed to lead, cadmium and arsenic, two subgroups were distinguished: those with abnormal values of TSH and with normal TSH. Using the criterion of 1st and 2nd tercile of TSH, three subgroups were distinguished: manifesting TSH <1st tercile, TSH between 1st and 2nd tercile and those with TSH ≥2nd tercile.

Statistical analysis was conducted using the software of STATISTICA 10 (StatSoft Polska). Distribution of the variables was tested using tests of Lilliefors and Shapiro-Wilk. In cases of independent quantitative variables manifesting normal distribution, the subsequent statistical analysis used the *t* test for unlinked variables or the univariable, parametric analysis of variance. In cases of variables manifesting distribution distinct than the normal one, analysis of independent quantitative variables employed the *U* test of Mann-Whitney or a non-parametric equivalent of analysis of variance, the Kruskal-Wallis test. Significant statistical differences between arithmetic means were estimated using the post hoc test of Newman-Keuls. For independent qualitative variables, the subsequent statistical analysis used the chi-square test of the highest credibility. For determination of relationships between studied variables, analysis of logistic regression was conducted. Results at the level of *p* < 0.05 were accepted as significant.

## Results

In the study group of persons occupationally exposed to lead, cadmium and arsenic, mean Cd-B amounted to 2.34 μg/l, mean As-U was 14.31 μg/g crea, mean Pb-B was 222.09 μg/l and mean ZnPP was 42.67 μg/dl. Toxicological parameters in the studied group of individuals occupationally exposed to lead, cadmium and arsenic are presented in Table 3.

No differences were disclosed in mean values of toxicological parameters between the subgroups of persons occupationally exposed to lead, cadmium and arsenic with TSH in and out of the accepted normal values. Toxicological parameters in the subgroups distinguished basing on the criterion of normal values of thyrotropic hormone in blood are presented in Table 4.

**Table 4:** Toxicological parameters in subgroups distinguished basing on the criterion of normative values of thyrotropic hormone in blood

	Abnormal TSH	Normal TSH	*p*
Cd-B (μg/l)^a^	4.54 ± 5.21	1.90 ± 2.60	0.277
Cd-B ≥ 1st quartile^b^	100.0	80.0	0.488
Cd-B ≥ median^b^	100.0	40.0	0.121
Cd-B ≥ 3rd quartile^b^	50.0	30.0	0.584
Cd-B > MAC^b^	50.0	10.0	0.166
As-U (μg/g crea)^a^	10.83 ± 7.82	14.59 ± 12.80	0.447
As-U ≥ 1st quartile^b^	57.1	77.3	0.231
As-U ≥ median^b^	28.6	52.3	0.227
As-U ≥ 3rd quartile^b^	14.3	26.1	0.487
As-U > MAC^b^	0.0	6.8	0.475
Pb-B (μg/l)^a^	217.88 ± 87.45	222.45 ± 144.94	0.930
Pb-B ≥ 1st quartile^b^	87.5	74.5	0.411
Pb-B ≥ median^b^	62.5	48.9	0.461
Pb-B ≥ 3rd quartile^b^	12.5	27.6	0.351
Pb-B > MAC^b^	0.0	4.3	0.552
ZnPP (μg/dl)^a^	37.00 ± 13.42	43.15 ± 29.88	0.567
ZnPP ≥ 1st quartile^b^	75.0	75.5	0.973
ZnPP ≥ median^b^	50.0	51.0	0.954
ZnPP ≥3rd quartile^b^	25.0	25.5	0.974
ZnPP ≥ MAC^b^	0.0	9.6	0.359

*As-U* concentration of arsenic in urine, *Cd-B* concentration of cadmium in blood, *Cu-B* concentration of copper in blood, *MAC* maximum admissible concentrations, *Pb-B* concentration of lead in blood, *TSH* thyrotropic hormone, *Zn-B* concentration of zinc in blood, *ZnPP* concentration of zinc protoporphyrin in blood

^a^Arithmetic mean ± standard deviation

^b^Percentage

Also, no differences were disclosed in mean values of toxicological parameters between subgroups of persons with TSH <1st tercile, TSH between the 1st and the 2nd tercile and those with TSH ≥2nd tercile. The toxicological parameters in subgroups distinguished on the basis of 1st and 2nd tercile of TSH are presented in Table 5.

**Table 5:** Toxicological parameters in subgroups distinguished basing on the criterion of values of the first and the second terciles of thyrotropic hormone concentrations in blood

	TSH < 1st tercile	TSH: 1st–2nd tercile	TSH ≥ 2nd tercile	*p*
Cd-B (μg/l)^a^	0.86 ± 0.00	3.05 ± 4.48	2.26 ± 2.82	0.841
Cd-B ≥ 1st quartile^b^	100.0	100.0	75.0	0.549
Cd-B ≥ median^b^	100.0	33.3	50.0	0.513
Cd-B ≥ 3rd quartile^b^	0.0	33.3	37.5	0.755
Cd-B > MAC^b^	0.0	33.3	12.5	0.638
As-U (μg/g crea)^a^	15.01 ± 12.13	13.38 ± 8.71	14.67 ± 16.11	0.862
As-U ≥ 1st quartile^b^	75.9	79.4	71.9	0.775
As-U ≥ median^b^	58.6	55.9	37.5	0.190
As-U ≥ 3rd quartile^b^	34.5	17.7	25.0	0.309
As-U > MAC^b^	3.5	5.9	9.4	0.631
Pb-B (μg/l)^a^	204.38 ± 147.94	255.45 ± 155.12	204.95 ± 114.11	0.098
Pb-B ≥ 1st quartile^b^	60.6	88.6	76.5	0.067
Pb-B ≥ median^b^	48.5	57.1	44.1	0.545
Pb-B ≥ 3rd quartile^b^	30.3	31.4	17.6	0.359
Pb-B > MAC^b^	3.0	8.6	0.0	0.177
ZnPP (μg/dl)^a^	41.09 ± 22.07	50.69 ± 41.84	35.94 ± 13.01	0.227
ZnPP ≥ 1st quartile^b^	75.8	80.0	70.6	0.661
ZnPP ≥ median^b^	54.6	57.1	41.2	0.366
ZnPP ≥3rd quartile^b^	27.3	28.6	20.6	0.719
ZnPP ≥ MAC^b^	9.1	17.1	0.0	0.053

*As-U* concentration of arsenic in urine, *Cd-B* concentration of cadmium in blood, *Cu-B* concentration of copper in blood, *MAC* maximum admissible concentrations, *Pb-B* concentration of lead in blood, *TSH* thyrotropic hormone, *Zn-B* concentration of zinc in blood, *ZnPP* concentration of zinc protoporphyrin in blood

^a^Arithmetic mean ± standard deviation

^b^Percentage

Logistic non-linear regression yielded the following model, taking into account anthropological parameters (age and BMI), biochemical parameters (glucose, total bilirubin, alanine and aspartate aminotransferases, creatinine and uric acid, total cholesterol, HDL cholesterol and triglycerides) and toxicological parameters (Cd-B, Pb-B, ZnPP and As-U):

logit abnormal TSH = 1.496 total bilirubin + 1.262 Cd-B

The obtained model demonstrated that higher blood total bilirubin concentrations and higher Cd-B represented independent risk factors of abnormal values of TSH in this group. Complete logistic regression analysis results are presented in Table 6.

**Table 6:** Results of logistic regression analysis

	Model for Probability of abnormal TSH			
	Regression coefficient	SEM of Rc	*p* value	Odds ratio (for unit change)
Intercept	1.376	6.566	0.834	3.960
Age (years)	−0.093	0.067	0.171	0.911
Body mass index (kg/m^2^)	0.024	0.141	0.863	1.025
Glucose (mg/dl)	−0.035	0.033	0.292	0.965
Total bilirubin (mg/dl)	1.496	5.038	0.025	4.101
Alanine aminotransferase (U/l)	0.042	0.044	0.343	1.043
Aspartate aminotransferase (U/l)	0.002	0.069	0.977	1.002
Creatinine (mg/dl)	0.173	4.425	0.969	1.189
Uric acid (mg/dl)	0.566	0.639	0.378	1.761
Total cholesterol (mg/dl)	0.010	0.017	0.571	1.010
High density lipoprotein (mg/dl)	−0.009	0.076	0.910	0.991
Triglycerides (mg/dl)	−0.001	0.003	0.844	0.999
Cd-B (μg/l)	1.262	0.562	0.027	1.532
As-U (μg/g crea)	−0.022	0.046	0.626	0.978
Pb-B (μg/l)	0.004	0.006	0.473	1.004
ZnPP (μg/dl)	−0.063	0.052	0.231	0.939

*As-U* concentration of arsenic in urine, *Cd-B* concentration of cadmium in blood, *Pb-B* concentration of lead in blood, *SEM of Rc* standard error of the regression coefficient, *TSH* thyrotropic hormone, *ZnPP* concentration of zinc protoporphyrin in blood

## Discussion

Analysis of results obtained in this study justifies the statement that in the group of workers exposed to lead, cadmium and arsenic, a relationship between blood cadmium concentration and blood thyrotropic hormone concentration exists, but this relation is a non-linear one. A further analysis has shown that in the studied group no relationships existed between the other toxicological parameters and blood thyrotropic hormone concentration. Comparative analyses did not reveal any statistically significant differences in toxicological parameters between subgroups of patients with different blood TSH concentrations. Nevertheless, in parallel, analysis of non-linear logistic regression has demonstrated that higher blood cadmium concentrations, aside from higher blood total bilirubin concentration, represented an independent risk factor of abnormal blood thyrotropic hormone concentrations.

The results of the present study may significantly supplement current knowledge on the relationship between exposure to metals and thyroid function. As abovementioned, the studies on the subject do not allow an unequivocal confirmation of such a relationship [5–7, 16, 17].

Lead represents one of the better examined heavy metals, as far as their effect on thyroid hormone concentrations is considered. Studies conducted on a population exposed to high lead concentrations (lead concentration in blood ≥50 μg/dl) suggested a negative relationship between lead concentration in blood and concentrations of T4, fT4 and T3 hormones [17–19]. Zadjali et al. also showed that the exposure of diabetic rats to lead acetate caused changes that are consistent with clinical hypothyroidism i.e. high TSH and low T4 and T3 levels [20]. In turn, studies independently conducted by e.g. Erfurth et al., Schumacher et al. and Refowitz et al., on a similar population detected no significant relationship between the variables [21–24]. Results of a few investigations are also available, in which the relationship was examined between lead concentration in blood and thyroid function in populations manifesting Pb-B <10 μg/dl. In such a population, Dundar et al. documented a negative correlation between Pb-B and fT4 [7]. Meeker et al. proved in turn a negative relationship between Pb-B and TSH [10], while Mendy et al. demonstrated a negative relationship between Pb-B and total thyroxin as well as absence of a significant correlation between Pb-B and TSH, TT3, fT3 and fT4 [25]. A similar absence of a significant relationship between Pb-B and concentration of thyroid hormones in blood was found by Chen et al. [26]. Abdelouahab et al. identified absence of a significant relationship between thyroid hormone concentrations and concentration of lead in men. On women, on the other hand, a positive relationship was disclosed between Pb-B and T3 and a negative one between Pb-B and TSH [27]. A similar conclusion resulted from studies conducted by Schell et al., who documented also a positive relationship between Pb-B and T3 (mean lead concentration of 1.3 ± 0.15 μg/dl); in turn, Singh detected a positive relationship between Pb-B and TSH (mean lead concentration of 51.9 ± 9.4 μg/dl) [28, 29]. The discussed studies seemed to exclude any relationship between concentrations of lead and zinc protoporphyrin in blood and concentration of thyrotropic hormone in a population exposed to lead, cadmium and arsenic.

Exposure to cadmium may induce either acute or chronic damage of several organs, first of all liver, kidneys, lungs, bones and testes. However, the available literature contains also certain data suggesting negative influence of cadmium on thyroid gland [6, 11, 26, 30–34]. Rasic-Milutinovic et al. showed a potential toxic effect of cadmium on thyroid function in Hashimoto thyroiditis patients [35]. Studies conducted on animals exposed to cadmium pointed to lowered concentrations of TT4 or TT4 and TT3 in serum [31–33]. The suggested mechanism explaining the described effect might involve e.g. inhibition of synthesis and/or release of thyroxin, a disturbed process of T4 deiodination due to inhibited activity of 5-deiodinase [33]. In studies of Hammoudy et al. on experimental animals, supplementation with selenium and zinc reduced the cadmium-induced thyroid dysfunction [31, 32]. Interesting were also results of studies by Gupta and Kar, who provided evidence for a protective effect exerted by administration of ascorbic acid, which reduced the cadmium-induced decrease in serum T3 concentration, reduction of hepatic deiodinase activity and augmented peroxidation of cell membrane lipids. The effect resulted from antioxidative effects of ascorbic acid [33]. Mohamed et al. demonstrated significantly increased levels of TSH in Cd-treated rats compared with the control group. The addition of phytic acid in diet decreased the high levels of TSH [36]. Chen et al., in studies performed within the National Health and Nutrition Examination Survey (NHANES) in 2007–2008 in USA on a group of around 5000 individuals, demonstrated a positive relationship between cadmium concentration in urine and concentrations of TT4, TT3, fT3 and thyroglobulin [26]. In turn, studies performed by Ijima et al. in groups of 24 women and 24 newborns showed that concentration of cadmium in umbilical blood showed a significant negative correlation with concentration of TSH in blood of the newborns [6]. Christensen et al. described a negative correlation between cadmium concentration in blood and concentration of TSH and a positive correlation between concentration of cadmium in urine and concentrations of T3 and fT3. No significant relationship was demonstrated between cadmium concentration in blood and concentration of TSH in studies of Meeker et al. conducted on 219 men, patients of an infertility clinic [10]. The abovementioned studies, as it was already accentuated, point to a non-linear relationship between elevated cadmium concentration in blood and abnormal concentrations of thyrotropic hormone in a population of individuals exposed to lead, cadmium and arsenic.

Epidemiological studies link chronic exposure to arsenic first of all to increased incidence of tumours, such as neoplasms of lungs, urinary bladder, skin and liver, and of several other diseases, including coronary artery disease, diabetes, infertility and neurological and dementive diseases [5, 37–40]. In the context of thyroid function, the available studies demonstrated that at the cellular level arsenic disturbs gene control taking place with mediation of steroid hormone receptors and receptors of retinoic acid and of thyroid hormones [5, 41, 42]. In turn, Palazzolo et al. confirmed the ability of arsenic trioxide to inhibit the activity of TPO in vitro [43]. Sun et al. showed that arsenic concentrations at ≤150 μg/l interfered with thyroid hormone homeostasis in bighead carp larvae by increasing thyroid hormone levels and reducing Trs mRNA transcriptional levels [44]. Additionally, it has been demonstrated that arsenite caused oxidative damage, affected thyroid endocrine system and altered gene transcription in the hypothalamic-pituitary-thyroid axis in zebrafish [45]. Investigations of Kotyzova et al. failed to demonstrate effects of selenium and iodine supplementation on storage of arsenic in rat thyroid glands (in cases of exposure to bromine supplementation with iodine and selenium induced 50% reduction of bromine uptake by thyroid gland). On the other hand, exposure to arsenic induced an increase in iodine concentration and a decrease in selenium concentration in thyroids of animals administered with iodine and selenium [46]. Meeker et al. described a positive relationship between arsenic concentration in blood and serum TSH concentration in 219 men, patients of an infertility clinic. The effect was dependent on the dose [10]. The discussed investigations seem to exclude a relationship between arsenic concentration in urine and concentration of thyrotropic hormone in the population of individuals exposed to lead, cadmium and arsenic.

The main limitation of the study is the absence of a control group composed of individuals who are not exposed to metals at the workplace, with similar clinical characteristics and comparable environmental exposure to metals, failure to measure the level of other thyroid hormones (fT3 and fT4) and a complex character of the exposure (parallel exposure to lead, cadmium and arsenic).

Summing up the above, the obtained results of investigations may prompt further studies related to relationships between exposure to various chemical agents and thyroid function, particularly in the context of parallel exposure to low concentrations of various substances. The data related to the relationship between exposure to lead, cadmium and arsenic remain incomplete, and, as proven also by this study, they are frequently equivocal or even contradictory. As far as exposure to other metals affects thyroid function, we have at our disposal only individual reports pertaining exposure to mercury, manganese, molybdenum, barium, thallium, caesium and uranium [8, 11]. Moreover, the idea to conduct population-based studies should be postulated, related to such relationships.

## Conclusion

1. In the group of workers exposed to lead, cadmium and arsenic, a relationship between blood cadmium concentration and blood thyrotropic hormone concentration has been documented, but this relation is a non-linear one.

2. In the group of workers exposed to lead, cadmium and arsenic, higher blood cadmium concentration seems to augment the risk of abnormal hormonal thyroid function.
